# Determination of Mercury in an Assortment of Dietary Supplements Using an Inexpensive Combustion Atomic Absorption Spectrometry Technique

**DOI:** 10.1155/JAMMC.2005.211

**Published:** 2005

**Authors:** Keith E. Levine, Michael A. Levine, Frank X. Weber, Ye Hu, Jason Perlmutter, Peter M. Grohse

**Affiliations:** 1 RTI International 3040 Cornwallis Road PO Box 12194 Research Triangle Park NC 27709 USA

## Abstract

The concentrations of mercury in forty, commercially available dietary supplements, were determined using a new, inexpensive analysis technique. The method involves thermal decomposition, amalgamation, and detection of mercury by atomic absorption spectrometry with an analysis time of approximately six minutes per sample. The primary cost savings from this approach is that labor-intensive sample digestion is not required prior to analysis, further automating the analytical procedure. As a result, manufacturers and regulatory agencies concerned with monitoring lot-to-lot product quality may find this approach an attractive alternative to the more classical acid-decomposition, cold vapor atomic absorption methodology. Dietary supplement samples analyzed included astragalus, calcium, chromium picolinate, echinacea, ephedra, fish oil, ginger, ginkgo biloba, ginseng, goldenseal, guggul, senna, St John's wort, and yohimbe products. Quality control samples analyzed with the dietary supplements indicated a high level of method accuracy and precision. Ten replicate preparations of a standard reference material (NIST 1573a, tomato leaves) were analyzed, and the average mercury recovery was 109% (2.0% RSD). The method quantitation limit was 0.3 ng, which corresponded to 1.5 ng/g sample. The highest found mercury concentration (123 ng/g) was measured in a concentrated salmon oil sample. When taken as directed by an adult, this product would result in an approximate mercury ingestion of 7 μg per week.


Journal of Automated Methods & Management in Chemistry, **2005** (2005), no. 4, 211–216
© 2005 Hindawi Publishing Corporation

### Determination of Mercury in an Assortment of Dietary Supplements Using an Inexpensive Combustion Atomic Absorption Spectrometry Technique

Keith E. Levine, Michael A. Levine, Frank X. Weber, Ye Hu, Jason Perlmutter, and Peter M. Grohse

*RTI International, 3040 Cornwallis Road, PO Box 12194, Research Triangle Park, NC 27709, USA*

*Received 15 July 2005; Accepted 20 July 2005*

The concentrations of mercury in forty, commercially available dietary supplements, were determined using a new, inexpensive analysis technique. The method involves thermal decomposition, amalgamation, and detection of mercury by atomic absorption spectrometry with an analysis time of approximately six minutes per sample. The primary cost savings from this approach is that labor-intensive sample digestion is not required prior to analysis, further automating the analytical procedure. As a result, manufacturers and regulatory agencies concerned with monitoring lot-to-lot product quality may find this approach an attractive alternative to the more classical acid-decomposition, cold vapor atomic absorption methodology. Dietary supplement samples analyzed included astragalus, calcium, chromium picolinate, echinacea, ephedra, fish oil, ginger, ginkgo biloba, ginseng, goldenseal, guggul, senna, St John's wort, and yohimbe products. Quality control samples analyzed with the dietary supplements indicated a high level of method accuracy and precision. Ten replicate preparations of a standard reference material (NIST 1573a, tomato leaves) were analyzed, and the average mercury recovery was 109% (2.0% RSD). The method quantitation limit was 0.3 ng, which corresponded to 1.5 ng/g sample. The highest found mercury concentration (123 ng/g) was measured in a concentrated salmon oil sample. When taken as directed by an adult, this product would result in an approximate mercury ingestion of 7 $\mu$g per week.

### 1. INTRODUCTION

The physical properties of mercury have given this toxic element and its compounds industrial utility throughout recorded history. Current uses of mercury include the manufacture of vapor lamps, barometers, switches, medical devices, chlorine (through the chlor-alkali process), and the extraction of gold from ore. An unfortunate consequence of this widespread industrial use has been human exposure. The most prevalent mercury exposure pathway for the general population is ingestion of contaminated food [1] with particularly acute toxicity for neonatal and fetal central nervous system tissues [2].

Dietary supplements are ingested regularly and constitute a growing component of the health care market. In the USA alone, $12 billion was spent on dietary supplements in the year 2000 with over 800 companies involved in the manufacture or distribution of these products [3]. The increasing popularity and use of dietary supplements has prompted numerous investigations into their quality and purity. Mercury contamination has been a focus area of several of these studies. In a recent investigation, approximately 10% of 500 Chinese patent medicinal products imported into the USA contained undeclared drugs or potentially toxic levels of heavy metals, including mercury [4]. In another study, 100 Malaysian herbal medicine preparations were tested for mercury, and 36 did not comply with Malaysian quality requirements [5]. Elevated mercury levels were also reported in some Asian herbal remedies tested in other investigations [6, 7, 8]. In contrast, the mercury levels in a number of recently tested Brazilian [9], Chinese [10], and South African [11] herbal remedies did not appear to be a public health concern. Mercury was not detected in an assortment of over-the-counter ginseng preparations from USA, Europe, and Asia [12], and a significant amount of mercury was not observed in tested fish oil preparations commercially available in the USA [13].

With the debate over the results of these investigations [14, 15], further product testing seems likely. A number of analytical tools are available to dietary supplement manufacturers and regulatory agencies for mercury contamination and adulteration measurements. The most frequently employed technique for the determination of mercury in botanical or similar matrices is cold vapor atomic absorption spectrometry (CVAAS). This analytical tool has been applied successfully to determine mercury levels in mosses,

Correspondence and reprint requests to Keith E. Levine, RTI International, 3040 Cornwallis Road, PO Box 12194, Research Triangle Park, NC 27709, USA; E-mail: levine@rti.org.

lichens, and aquatic plants [16, 17, 18], rice grain samples [19], mushrooms [20, 21, 22], and food samples of a botanical origin [23, 24]. Other techniques include inductively coupled plasma optical emission spectrometry (ICP-OES) and inductively coupled plasma mass spectrometry (ICP-MS) [25], electrothermal atomic absorption spectrometry (ETAAS) [26], cold vapor atomic fluorescence spectrometry (CVAFS) [27], instrumental neutron activation analysis (INAA) [28], X-ray diffraction (XRD) [29], and X-ray fluorescence (XRF) spectrometry [30].

Application of these instrumental tools for the determination of mercury in dietary supplements may be cost prohibitive for many manufacturers. Perhaps the most significant cost associated with several of the aforementioned techniques is labor-intensive sample decomposition prior to analysis. A method for the determination of mercury that requires minimal sample pretreatment is thermal decomposition, amalgamation, and detection by atomic absorption spectrometry. Using this methodology, a direct mercury analysis can be completed for a dietary supplement sample in approximately six minutes. This direct analysis approach recently proved to be successful for completing mercury measurements in the coal, fly ash, bottom ash, and flue gas desulfurization material from coal-fired power plants [31] and in solid peat samples [32]. A similar methodology utilizing a pyrolysis unit coupled with atomic absorption spectrometry determined the concentration of mercury in three Chinese medicinal herbs [33].

The goal of this investigation was to measure the mercury content in an assortment of dietary supplements by applying this inexpensive direct mercury analysis methodology with minimal sample pretreatment. Purchased products included both plant and animal product-based dietary supplements labeled to enhance human health functions.

### 2. EXPERIMENTAL

### 2.1. *Study samples and reagents*

An assortment of forty commercially available, over-the-counter dietary supplements was purchased for mercury analysis. The assortment included astragalus (one sample), calcium (three samples), chromium picolinate (three samples), echinacea (two samples), ephedra (two samples), fish oil (three samples), ginger (three samples), ginkgo biloba (four samples), ginseng (four samples), goldenseal (three samples), guggul (one sample), senna (five samples), St John's wort (three samples), and yohimbe (three samples) products. Each product within a dietary supplement classification was purchased from a different manufacturer. It is important to note that only one lot of each product was purchased. The ephedra products were purchased prior to the United States Food and Drug Administration's (USFDA) recent ban on these supplements.

Several standard reference materials (SRM) and certified reference materials (CRM) from the National Institute of Standards and Technology (NIST) and the National Research Council of Canada (NRCC) were used throughout this investigation. San Joaquin soil (NIST SRM 2709) was used for instrument calibration, dogfish muscle tissue (NRCC DORM-1) was utilized to prepare matrix spike samples, and lobster hepatopancreas tissue (NRCC TORT-2) was used as a calibration check. Aliquots of tomato leaves (NIST SRM 1573a) were also analyzed with each sample batch to assess method accuracy. The certified mercury concentrations of the San Joaquin soil, dogfish muscle tissue, lobster hepatopancreas tissue, and tomato leaves were $1400 \pm 80$ ng/g, $798 \pm 74$ ng/g, $270 \pm 60$ ng/g, and $34 \pm 4$ ng/g, respectively.

Trace metal grade nitric acid ($HNO_3$) from Fisher Scientific (Pittsburgh, Pa) and type I quality deionized (DI) water from Pure Water Solutions (Hillsborough, NC) were used to clean plastic sample storage bottles.

### 2.2. *Labware preparation*

Plastic storage bottles were acid-cleaned prior to use for this investigation to minimize the potential for mercury contamination. The bottles and caps were rinsed several times with DI water and placed in a 20% (v/v) $HNO_3$ bath for overnight leaching. On the following day, bottles and caps were removed from the bath, rinsed several times with DI water, and dried under HEPA-filtered air. If not used immediately, the bottles were capped and sealed in plastic storage bags.

### 2.3. *Sample preparation*

Because the employed instrumental technique does not require digestion prior to analysis, labor-intensive sample preparation was not required. For each solid product encapsulated in gelatin, the content of approximately one third of the capsules was transferred to an acid-cleaned plastic storage bottle. One gelatin capsule was randomly chosen from each of the fish oil products and punctured with a metal-free spatula just prior to analysis. The content of the ruptured capsule was then transferred directly to a nickel sample boat. For each product purchased in tablet form, approximately one third of the tablets were manually ground into a fine powder. The ground powder was then transferred to an acid-cleaned plastic storage bottle.

### 2.4. *Mercury analysis*

Sample analysis was completed using a DMA-80 direct mercury analyzer (Milestone, Bergamo, Italy). A nominal 200 mg aliquot of each dietary supplement sample (100 mg nominal aliquot for fish oil samples) was weighed directly into a nickel boat and the boat was placed in the instrument autosampler tray. The autosampler then inserted each sample into an oxygenated decomposition furnace to liberate mercury. Within the instrument, the samples were subjected to drying and decomposition steps, and the combustion products were carried through a heated catalyst that converted different mercury species to elemental mercury vapor. This mercury vapor then traveled to the system amalgamator where it was trapped. After a system flush with oxygen to remove any remaining non-mercury vapor, the amalgamator was heated to release trapped mercury. An oxygen stream carried the mercury vapor into the instrument's absorbance cell where it was

Table 1: Milestone DMA-80 instrumental parameters.

| | |
|---|---|
| Drying temperature | 300 °C |
| Drying time | 60 s |
| Decomposition temperature | 850 °C |
| Decomposition time | 180 s |
| Wait time | 60 s |
| Amalgam heating time | 12 s |
| Measurement time | 30 s |

positioned in the path of a single wavelength atomic absorption spectrometer. The absorption peak area, measured at 253.7 nm, was then used as a measure of mercury concentration. This process is described in greater detail in United States Environmental Protection Agency (USEPA) Method 7473 for the analysis of mercury by thermal decomposition, amalgamation, and atomic absorption spectrometry [34]. The DMA-80 temperature and time parameters employed in this investigation are presented in Table 1.

Prior to sample analysis, the instrument was calibrated with aliquots of San Joaquin soil (NIST SRM 2709). Soil masses were selected so that the nominal mercury level in the calibration standards would range from 0–600 ng Hg. For the purpose of analysis, the instrument generated two calibration curves. The first curve ranged from 0–40 ng Hg, while the second curve ranged from 40–600 ng Hg. This approach allowed for more accurate measurements at lower mercury concentrations without shortening the overall mercury working range.

Several quality control (QC) measures were taken during the DMA-80 mercury analyses. Instrument and calibration performance was evaluated prior to sample analysis on each operation day. Low (nominal 15.0 mg), mid (nominal 75.0 mg), and high (nominal 150 mg) aliquots of lobster hepatopancreas tissue (NRCC TORT-2) were analyzed for nominal mercury content of 4.05 ng, 20.3 ng, and 40.5 ng, respectively. Calibration checks were considered accurate if the determined mercury concentrations for all three TORT-2 samples fell within the confidence levels reported on the CRM certificate of analysis (270 ± 60 ng/g). In addition to this calibration check, a blank boat and at least one TORT-2 aliquot were analyzed before and after each group of ten samples. For the analysis of bracketed samples to be considered valid, the total mercury content measured from the blank cuvette was required to be less than 0.15 ng, and the determined TORT-2 concentration was required to be within ±25% of its certified concentration.

Several other QC samples were prepared and analyzed concurrently with the dietary supplement samples to further assess method performance. Ten replicate aliquots of a botanical SRM (NIST SRM 1573a; tomato leaves) were analyzed with the samples as a measure of method accuracy. In addition, six of the dietary supplement samples were selected randomly and analyzed in duplicate to assess method precision. Finally, four dietary supplement samples were fortified with aliquots of dogfish muscle tissue (NRCC DORM-1). These matrix spike samples were prepared and analyzed to determine the recovery of mercury in the presence of sample matrix.

### 3. RESULTS AND DISCUSSION

### 3.1. Method quantitation limit

During the course of this investigation, ten empty nickel boats were analyzed as components of QC sample sets and were treated as blanks. The method quantitation limit (MQL) was defined as ten times the standard deviation of the total mercury level (in ng) measured in these blank runs. The average measured mercury level for the blanks was 0.049 ng, and the determined MQL for mercury was 0.3 ng, which can be expressed as 1.5 ng/g by assuming a nominal 0.2 g sample mass.

### 3.2. Instrument calibration

Prior to sample analysis, the instrument was calibrated using a soil SRM, and two curves were constructed. The regression equation for the low-level mercury curve (0–40 ng Hg) was absorbance $= 0.02891(x) - 0.00019(x^2)$, while the regression equation for the high-level mercury curve (40–600 ng Hg) was absorbance $= 0.00168(x)$. For each of three sample analysis batches, at least three TORT-2 CRM samples were used to check instrument calibration. The determined mercury concentration for these check samples ranged from 277–323 ng/g, corresponding to 103–120% recovery of the certified mercury value (270 ± 60 ng/g). The determined mercury concentrations for all of the calibration check standards fell within the uncertainty range specified on the TORT-2 CRM certificate of analysis.

### 3.3. Accuracy and precision

Ten replicate aliquots of a botanical SRM (NIST SRM 1573a; tomato leaves) were analyzed along with the dietary supplement samples during this investigation. The average mercury concentration was determined to be 36.9 ng/g, or 109% of the certified value (34 ± 4 ng/g). The determined mercury concentration for each replicate was within the uncertainty range specified on the SRM certificate of analysis. The percent relative standard deviation (RSD) for these replicates was 2.0%. The high level of accuracy and precision demonstrated by the replicate analyses of this botanical SRM indicate acceptable method performance.

### 3.4. Matrix impact

Four dietary supplement samples were fortified with aliquots of the dogfish muscle tissue CRM (NRCC DORM-1) to determine the recovery of mercury in the presence of sample matrix. A nominal 30 mg aliquot of the CRM was added to each of four 200 mg aliquots of dietary supplement samples. Mercury spikes from the added CRM were recovered in yohimbe, goldenseal, guggul, and echinacea matrices at 114%, 107%, 120%, and 116%, respectively. In each instance, the determined concentration of mercury fell within the uncertainty range listed on the CRM certificate of analysis.

Table 2: Mercury content of tested dietary supplements in ng/g.

| Product name | ng Hg/g | Product name | ng Hg/g |
|---|---|---|---|
| Astragalus product 1 | 7.41 (5.1%)[a] | Goldenseal product 1 | 15.9 (6.7%) |
| Calcium product 1 | < MQL[b] | Goldenseal product 2 | 13.8 |
| Calcium product 2 | 1.52 | Goldenseal product 3 | 8.81 |
| Calcium product 3 | < MQL | Guggul product 1 | 1.68 |
| Chromium picolinate product 1 | 1.66 | Senna product 1 | 16.7 |
| Chromium picolinate product 2 | < MQL | Senna product 2 | 14.3 |
| Chromium picolinate product 3 | < MQL | Senna product 3 | 12.3 |
| Ginger product 1 | 14.9 (2.4%) | Senna product 4 | 1.81 |
| Ginger product 2 | 27.4 | Senna product 5 | 12.1 |
| Ginger product 3 | 10.8 | Echinacea product 1 | 2.11 |
| Ginkgo biloba product 1 | 2.94 | Echinacea product 2 | 34.9 (12%) |
| Ginkgo biloba product 2 | 32.0 (1.3%) | Fish oil product 1 | 123 |
| Ginkgo biloba product 3 | 5.76 | Fish oil product 2 | 38.8 |
| Ginkgo biloba product 4 | 79.0 | Fish oil product 3 | 9.89 |
| Ginseng product 1 | 15.3 | St John's wort product 1 | 3.86 |
| Ginseng product 2 | 13.8 | St John's wort product 2 | 2.47 |
| Ginseng product 3 | 5.53 | St John's wort product 3 | 2.27 |
| Ginseng product 4 | 6.22 | Yohimbe product 1 | 13.3 |
| Ephedra product 1 | 3.58 | Yohimbe product 2 | 3.28 |
| Ephedra product 2 | 5.30 (5.3%) | Yohimbe product 3 | 4.16 |

[a]Product prepared and analyzed in duplicate; %RSD provided in parentheses.
[b]Determined product concentration less than < MQL (1.5 ng/g).

Overall, the observed recoveries were similar for each test matrix, suggesting no significant matrix impact.

### 3.5. *Mercury content of dietary supplements*

Mercury data for the assortment of commercially available, over-the-counter dietary supplements are presented in Table 2. Six samples (astragalus product 1, ginger product 1, ginkgo biloba product 2, ephedra product 2, goldenseal product 1, and echinacea product 2) were analyzed in duplicate. The %RSD data provided in the table for these six samples indicate a high degree of precision in the sample weighing and analysis procedures.

### 4. CONCLUSION

The objective of this investigation was to determine the mercury content in an assortment of forty, commercially available dietary supplements by applying an inexpensive, direct mercury analysis methodology. The primary cost savings from this approach is that labor-intensive sample digestion is not required, making the technique more automated and less expensive than conventional mercury analysis methods. The reduced sample handling also helps to minimize the potential for mercury contamination.

A review of the data presented in Table 2 reveals that the tested calcium, chromium picolinate, ephedra, guggul, and St John's wort products all had relatively low measured mercury levels when compared to the other tested supplements. The highest mercury levels were observed for some of the ginkgo biloba, fish oil, and echinacea products. Overall, fish oil product 1 had the highest measured mercury concentration (123 ng/g). The recommended dose of this product, which was labeled as a salmon oil concentrate, was two capsules, three times daily. When taken as directed, this product would result in approximate mercury ingestion of 7 $\mu$g per week. A joint World Health Organization (WHO)/ Food and Agriculture Organization (FAO) expert committee recently lowered the provisional tolerable weekly intake (PTWI) of methylmercury to 1.6 $\mu$g/kg body weight per week [35]. If all of the mercury content of fish oil product 1 were in the form of methylmercury, a 63 kg person would still be below his or her 100 $\mu$g/week PTWI. When taken as directed, the other products tested in this investigation would not contribute significantly to the PTWI.

Several instances where the mercury content was highly variable for different products of the same dietary supplement were observed. For example, the determined mercury concentrations of ginkgo biloba products 2 and 4 were 32.0 ng/g and 79.0 ng/g, respectively, while the determined concentrations for ginkgo biloba products 1 and 3 were 2.94 ng/g and 5.76 ng/g, respectively. This variability may reflect different environmental conditions at harvest or different processing procedures used by the manufacturers. It is important to note that only one lot of each product was tested. As a result, the mercury data in this manuscript do not reflect potential lot-to-lot product variability.

When the toxicity of mercury is coupled with the potential variability in the mercury content of dietary supplements, additional product testing seems likely. Method sensitivity, accuracy, and precision indicate that the described methodology could be applied successfully to the determination of mercury in a wide variety of dietary supplement samples to meet this testing need. The described analysis would be less expensive than more established mercury measurement techniques, such as CVAAS, because labor-intensive sample digestion is not required. The lower measurement cost could allow for more screens by dietary supplement manufacturers and regulatory agencies that may otherwise have been cost prohibitive. It is important to note that the methodology employed during this investigation was specific for the determination of mercury. If it is necessary to determine the concentration of other trace elements, sample digestion would be required.

### REFERENCES

[1] L. Järup, "Hazards of heavy metal contamination," *British Medical Bulletin*, vol. 68, no. 1, pp. 167–182, 2003.

[2] P. M. Rodier, "Vulnerable periods and processes during central nervous system development," *Environmental Health Perspectives*, vol. 102, no. 1, pp. 121–124, 1994.

[3] C. M. Foley and A. M. Kratz, "Resources and guidelines on buying and using nutraceuticals," in *Nutraceuticals: the Complete Encyclopedia of Supplements, Herbs, Vitamins, and Healing Foods*, A. J. Roberts, M. E. O'Brien, and G. Subak-Sharpe, Eds., pp. 635–647, Penguin Putnam, New York, NY, USA, 2001.

[4] A. M. Au, R. Ko, F. O. Boo, R. Hsu, G. Perez, and Z. Yang, "Screening methods for drugs and heavy metals in Chinese patent medicines," *Bulletin of Environmental Contamination and Toxicology*, vol. 65, no. 1, pp. 112–119, 2000.

[5] H.-H. Ang, E.-L. Lee, and H.-S. Cheang, "Determination of mercury by cold vapor atomic absorption spectrophotometer in Tongkat Ali preparations obtained in Malaysia," *International Journal of Toxicology*, vol. 23, no. 1, pp. 65–71, 2004.

[6] I.-C. Chuang, K.-S. Chen, Y.-L. Huang, P.-N. Lee, and T.-H. Lin, "Determination of trace elements in some natural drugs by atomic absorption spectrometry," *Biological Trace Element Research*, vol. 76, no. 3, pp. 235–244, 2000.

[7] M. K. Wong and L. L. Koh, "Mercury, lead, and other heavy metals in Chinese medicines," *Biological Trace Element Research*, vol. 10, pp. 91–97, 1986.

[8] G. J. Garvey, G. Hahn, R. V. Lee, and R. D. Harbison, "Heavy metal hazards of Asian traditional remedies," *International Journal of Environmental Health Research*, vol. 11, no. 1, pp. 63–71, 2001.

[9] E. D. Caldas and L. L. Machado, "Cadmium, mercury and lead in medicinal herbs in Brazil," *Food and Chemical Toxicology*, vol. 42, no. 4, pp. 599–603, 2004.

[10] M. K. Wong, P. Tan, and Y. C. Wee, "Heavy metals in some Chinese herbal plants," *Biological Trace Element Research*, vol. 36, pp. 135–142, 1993.

[11] V. Steenkamp, M. von Arb, and M. J. Stewart, "Metal concentrations in plants and urine from patients treated with traditional remedies," *Forensic Science International*, vol. 114, no. 2, pp. 89–95, 2000.

[12] I. A. Khan, J. Allgood, L. A. Walker, E. A. Abourashed, D. Schlenk, and W. H. Benson, "Determination of heavy metals and pesticides in ginseng products," *Journal of AOAC International*, vol. 84, no. 3, pp. 936–939, 2001.

[13] S. E. Foran, J. G. Flood, and K. B. Lewandrowski, "Measurement of mercury levels in concentrated over-the-counter fish oil preparations: is fish oil healthier than fish?" *Archives of Pathology and Laboratory Medicine*, vol. 127, no. 12, pp. 1603–1605, 2003.

[14] N. Hohmann and K. Koffler, "Letters to the editor," *International Journal of Environmental Health Research*, vol. 12, no. 1, pp. 99–100, 2002.

[15] G. Garvey, R. Harbison, and R. Lee, "Letter to the editor," *International Journal of Environmental Health Research*, vol. 12, pp. 101–101, 2002.

[16] C. F. Calasans and O. Malm, "Elemental mercury contamination survey in a chlor-alkali plant by the use of transplanted Spanish moss, *Tillandsia usneoides* (L.)," *Science of The Total Environment*, vol. 208, no. 3, pp. 165–177, 1997.

[17] M. Kamal, A. E. Ghaly, N. Mahmoud, and R. Côté, "Phytoaccumulation of heavy metals by aquatic plants," *Environment International*, vol. 29, no. 8, pp. 1029–1039, 2004.

[18] M. V. Balarama Krishna, D. Karunasagar, and J. Arunachalam, "Study of mercury pollution near a thermometer factory using lichens and mosses," *Environmental Pollution*, vol. 124, no. 3, pp. 357–360, 2003.

[19] I. Al-Saleh and N. Shinwari, "Report on the levels of cadmium, lead, and mercury in imported rice grain samples," *Biological Trace Element Research*, vol. 83, no. 1, pp. 91–96, 2001.

[20] J. Falandysz, L. Bielawski, K. Kannan, M. Gucia, K. Lipka, and A. Brzostowski, "Mercury in wild mushrooms and underlying soil substrate from the great lakes land in Poland," *Journal of Environmental Monitoring*, vol. 4, no. 4, pp. 473–476, 2002.

[21] J. Falandysz and A. Chwir, "The concentrations and bioconcentration factors of mercury in mushrooms from the Mierzeja Wislana sand-bar, Northern Poland," *Science of The Total Environment*, vol. 203, no. 3, pp. 221–228, 1997.

[22] J. Falandysz, M. Gucia, A. Brzostowski, et al., "Content and bioconcentration of mercury in mushrooms from Northern Poland," *Food Additives & Contaminants*, vol. 20, no. 3, pp. 247–253, 2003.

[23] O. Zenebon, A. M. Sakuma, S. Dovidauskas, I. A. Okada, F. D. de Maio, and J. Lichtig, "Rapid food decomposition by $H_2O_2$-$H_2SO_4$ for determination of total mercury by flow injection cold vapor atomic absorption Spectrometry," *Journal of AOAC International*, vol. 85, no. 1, pp. 149–152, 2002.

[24] R. Jedrzejczak, "Determination of total mercury in foods of plant origin in Poland by cold vapour atomic absorption spectrometry," *Food Additives & Contaminants*, vol. 19, no. 10, pp. 996–1002, 2002.

[25] J. Falandysz, K. Szymczyk, H. Ichihashi, et al., "ICP/MS and ICP/AES elemental analysis (38 elements) of edible wild mushrooms growing in Poland," *Food Additives & Contaminants*, vol. 18, no. 6, pp. 503–513, 2001.

[26] I. Karadjova and T. Venelinov, "Determination of arsenic and mercury in sunflower oil by electrothermal atomic absorption," *Food Additives & Contaminants*, vol. 19, no. 10, pp. 948–953, 2002.

[27] A. Göthberg, M. Greger, and B.-E. Bengtsson, "Accumulation of heavy metals in water spinach (*ipomoea aquatica*) cultivated in the Bangkok region, Thailand," *Environmental Toxicology and Chemistry*, vol. 21, no. 9, pp. 1934–1939, 2002.

[28] D. J. Samudralwar and A. N. Garg, "Minor and trace elemental determination in the Indian herbal and other medicinal preparations," *Biological Trace Element Research*, vol. 54, pp. 113–121, 1996.

[29] A. D. Hardy, H. H. Sutherland, R. Vaishnav, and M. A. Worthing, "A report on the composition of mercurials used in traditional medicines in Oman," *Journal of Ethnopharmacology*, vol. 49, no. 1, pp. 17–22, 1995.

[30] E. O. Espinoza, M. J. Mann, B. Bleasdell, S. DeKorte, and M. Cox, "Toxic metals in selected traditional Chinese medicinals," *Journal of Forensic Sciences*, vol. 41, no. 3, pp. 453–456, 1996.

[31] H. M. Boylan, R. D. Cain, and H. M. Kingston, "A new method to assess mercury emissions: a study of three coal-fired electric-generating power station configurations," *Journal of Air & Waste Management Association*, vol. 53, no. 11, pp. 1318–1325, 2003.

[32] F. Roos-Barraclough, N. Givelet, A. Martinez-Cortizas, M. E. Goodsite, H. Biester, and W. Shotyk, "An analytical protocol for the determination of total mercury concentrations in solid peat samples," *The Science of The Total Environment*, vol. 292, no. 1–2, pp. 129–139, 2002.

[33] C. Bin, W. Xiaoru, and F. S. C. Lee, "Pyrolysis coupled with atomic absorption spectrometry for the determination of mercury in Chinese medicinal materials," *Analytica Chimica Acta*, vol. 447, no. 1–2, pp. 161–169, 2001.

[34] United States Environmental Protection Agency, Method 7473, 1998.

[35] World Health Organization, Food and Agriculture Organization Joint Expert Committee on Food Additives, JECFA/61/SC:1-22, 2003.

